# The subunit vaccine of Lumpy skin disease virus elicits significant humoral and cell-mediated immune responses in mice

**DOI:** 10.3389/fvets.2025.1731692

**Published:** 2025-12-10

**Authors:** Xiaoxiao Gu, Xiaoyu Deng, Aodi Wu, Honghuan Li, Ziwei Liu, Wenxing Wang, Zhongchen Ma, Chuangfu Chen

**Affiliations:** 1 College of Animal Science and Technology, Shihezi University, Shihezi, Xinjiang, China; 2 Huaihua Key Laboratory of Ion Channels and Complex Diseases, School of Basic Medicine, Hunan University of Medicine, Changsha, Hunan, China

**Keywords:** LSDV, subunit vaccine, evaluation of immunogenicity, mouse, prokaryotic expression

## Abstract

Lumpy skin disease (LSD) is an emerging systemic and infectious disease affecting cattle. Currently, there is no specific treatment for this disease, and vaccination to boost immunity remains the most direct and effective approach for preventing LSD. In this study, we selected ORF073, ORF075, ORF090, and ORF110 proteins from the Lumpy skin disease virus (LSDV), which exhibit dominant antigenic properties, to construct, express, and identify recombinant prokaryotic expression vectors. The purified proteins were used to immunize mice. The immune efficacy of the subunit vaccines was preliminarily evaluated by monitoring antibody secretion and the expression of immune-related genes. The results showed that mice immunized with ORF075 and ORF090 subunit vaccines produced higher levels of antibody responses and induced a Th1/Th2-biased immune response. Splenocytes from immunized mice, when stimulated with vaccine antigens *in vitro*, exhibited the induction of higher levels of T-cell immune responses. These findings demonstrate that the LSDV ORF075 and ORF090 subunit vaccine developed in this study successfully induced robust humoral and cellular immune responses in mice, thereby providing a data foundation for subsequent experiments in the natural bovine host.

## 1 Introduction

Lumpy skin disease (LSD) is an acute, subacute, or chronic highly contagious infectious disorder afflicting cattle, caused by the Lumpy skin disease virus (LSDV). The disease is characterized by high fever, emaciation, swelling of superficial lymph nodes, lacrimation, conjunctivitis, and the appearance of prominent nodules on the skin, as well as on the mucous membranes of the oral cavity, respiratory tract, and genitalia (1). The principal mode of transmission is via blood-sucking arthropod vectors, which multiply and disperse in response to seasonal climatic variations (2). Moreover, the growing volume of international trade and the convenience of modern transportation have accelerated the spread of the disease, increasing the risk of its global dissemination. Consequently, there is an urgent imperative to develop novel and effective surveillance and prevention strategies to control this disease.

LSDV is a poxvirus with dimensions of approximately 270 nm × 290 nm. It has only one serotype and lacks hemagglutination activity (3, 4). The LSDV genome has an overall length of 145–156 kbp. It consists of a central coding region flanked by two identical 2.4 kbp inverted terminal repeat regions (5). The genome has an A+T content of 73% and contains 156 putative genes (6).

The disease is highly infectious and spreads widely, yet there are no effective therapeutic drugs available. Currently, in China, no homologous vaccine exists for this disease, and emergency immunization of cattle mainly depends on heterologous live-attenuated vaccines. These heterologous vaccines are derived from Goat pox virus (GTPV) and sheep pox virus (SPPV). However, due to the host specificity of GTPV and SPPV, there is a potential risk of infecting sheep and goats (7). Moreover, the use of live-attenuated vaccines may lead to adverse reactions, like the formation of nodules at the injection site. Therefore, there is an urgent necessity to develop a novel, highly efficient, and stable vaccine for the prevention and control of Lumpy Skin Disease in cattle.

ORF073, ORF075, ORF090, and ORF110 are homologs of poxvirus proteins that participate in virion morphogenesis and assembly, play a pivotal role in the pathogenesis of LSDV. They regulate viral structure and assembly, modulate the immune system's responses, and are involved in nucleotide biosynthesis (8, 9). In light of this, the present study employs LSDV structural and transmembrane proteins as potential vaccine candidates to screen immunodominant antigens, thus laying the foundation for the subsequent development of safe and effective LSDV vaccine candidates.

## 2 Materials and methods

### 2.1 Animal and bacterial strains

Female BALB/c mice, 4–6 weeks old, were procured from Ziqi Biological Company in Urumqi, Xinjiang, China. The animal study protocol received approval from the Animal Ethics Committee of Shihezi University, Xinjiang (Approval No.: A2025-013). All possible steps were taken to minimize animal suffering and reduce the number of animals used in the experiments.

The *Escherichia coli* strain BL21 (DE3) was employed for recombinant plasmid protein expression. The bacteria were cultivated in Luria–Bertani (LB) medium supplemented with antibiotics as necessary.

### 2.2 Evaluation of physicochemical properties, solubility, antigenicity, immunogenicity, allergenicity, and toxicity of proteins

The Expasy ProtParam server (https://web.expasy.org/protparam/) was utilized to predict the physicochemical properties of the subunit vaccines (10). For predicting the solubility of the designed subunit vaccines, the Protein-Sol server (https://protein-sol.manchester.ac.uk/) was employed (11). To assess the antigenicity and immunogenicity of the constructed vaccines, the ANTIGENpro server (https://scratch.proteomics.ics.uci.edu/) (12) and the IEDB Immunogenicity server (http://tools.iedb.org/immunogenicity/) (13) were utilized. Subsequently, AllerScreener (https://www.ddg-pharmfac.net/AllerScreener/screen/) and the ToxinPred server (https://webs.iiitd.edu.in/raghava/toxinpred2/) (14) were used to predict the allergenicity and toxicity of the vaccines, respectively.

### 2.3 Tertiary structure validation

The amino-acid sequences of the protein peptides were submitted to the relevant tool, and high-quality 3D structural models were obtained using AlphaFold2 (https://cryonet.ai/af2/). To validate the refined tertiary structures, Ramachandran plots were generated via the SWISS-MODEL workspace. Moreover, ProSA-web was utilized for the final validation of the vaccine protein structures.

### 2.4 Subunit proteins using *E coli* with explanation

The gene sequences of ORF073, ORF075, ORF090, and ORF110 were retrieved from LSDV XJ201901 (OM984485.1). Following codon optimization, and cloned into an ampicillin-resistant pCold-II vector (Tsingke Biotechnology, CN). The resulting plasmids were then transformed into *Escherichia coli* BL21 (DE3), Protein expression was induced with 0.5 mM IPTG at 16 °C for 20 h.

The proteins within the precipitate were purified according to the previously reported method (15). The purified proteins were characterized by SDS-PAGE. For the Western blot analysis, the mouse anti-His tag monoclonal primary antibody (1:1,000; Proteintech, Wuhan, China) and the goat anti-mouse IgG-HRP (H + L) antibody (1:1,000; Proteintech, Wuhan, China) were used. Protein concentrations were determined using a BCA protein assay kit (Solarbio, CN) by measuring the absorbance at 562 nm.

### 2.5 Vaccine preparation and mouse immunization protocol

Female SPF BALB/c mice (6 weeks old) were randomly allocated into different groups. A total of 60 mice were allocated into four candidate protein groups and one PBS control group (12 mice per group). For the first vaccination, the protein was mixed 1:1 with complete Freund's adjuvant (Sigma, CN) in PBS buffer, while for the second vaccination, it was mixed with incomplete Freund's adjuvant (Sigma, CN). Each mouse received a subcutaneous injection of 0.2 ml (100 μg protein) of the vaccine in two immunization rounds, which were conducted on day 0 and 14, respectively. As the negative control, PBS mixed with adjuvant was administered. Blood was collected from the mouse tails for the antibody response detection at weeks 0, 1, 2, 3, 4, 5, and 6. At 2 and 4 weeks after the last vaccination, spleens of three immunized mice were randomly collected to assess the cellular immune responses.

### 2.6 Detection of serum antibody titers by ELISA

The coating antigen (0.25 μg/ml, 1 μg/ml, 2 μg/ml, 4 μg/ml), test serum (1:50, 1:100, 1:500, 1:1,000), and HRP-conjugated goat anti-mouse IgG, IgG1, and IgG2a antibodies (Proteintech, CN; 1:10,000, 1:20,000, 1:50,000). The results were evaluated based on the P/N ratio, where a value ≥2.1 was considered positive. The P/N ratio was calculated as the OD value of the positive serum (P) divided by the OD value of the negative serum (N). The antigen coating concentration, serum dilution, and secondary antibody dilution corresponding to the highest P/N ratio were selected as the optimal conditions. Using these optimized parameters, an indirect ELISA was established to measure the levels of specific antibodies (IgG, IgG1, and IgG2a) in serum samples collected from mice immunized with various vaccine formulations at 7, 14, 21, 28, 35, and 42 days post-immunization. The IgG2a/IgG1 ratio was subsequently calculated.

### 2.7 Virus neutralization

Mouse serum samples were serially diluted two-fold and co-incubated with LSDV (200 TCID_50_) at 37 °C for 1 h. The virus-serum mixtures were then transferred to 96-well plates pre-seeded with 10,000 MDBK cells per well, with uninfected cell controls included. The cultures were maintained at 37 °C 5% CO_2_ for 5–7 days, with daily observation for CPE. The neutralizing antibody titer was determined using the Reed–Muench method.

### 2.8 Isolation of lymphocytes

At 28 days and 42 days post-immunization, BALB/c mice were euthanized by cervical dislocation, followed by disinfection via immersion in 75% ethanol. The spleens were aseptically excised and dissected to remove surrounding fascia. After weighing, the spleens were minced and gently ground through a 70 μm cell strainer. The resulting cell suspension was collected into 15 ml centrifuge tubes and centrifuged; the supernatant was discarded, and the pellet was resuspended for further processing.

Splenic lymphocytes were isolated with a commercial isolation kit for mice (TBD, CN). Lymphocyte separation solution was added to centrifuge tubes, and the splenic single-cell suspension was carefully layered on top. Following centrifugation 600 × g for 37 °C, the milky white lymphocyte layer was transferred to a new centrifuge tube. Washing buffer was added to the tube, and the cells were centrifuged at 250 × g for 37 °C; the supernatant was discarded. This washing procedure was repeated. Finally, the cells were resuspended in RPMI-1640 medium supplemented with 10% FBS, diluted appropriately, and subjected to cell counting. Based on the counting results, the cell concentration was adjusted to 1 × 106 cells/ml.

### 2.9 Elispot for measuring the IFN-γ in the immunized splenic lymphocytes

Specific T cell responses were detected by quantifying IFN-γ production in mouse splenocytes at 28 days and 42 days post-immunization using ELISpot assay. IFN-γ was detected using a mouse enzyme-linked immunospot (ELISPOT) kit (MABTECH, CN) following the manufacturer's instructions. A total of 2 × 106 freshly isolated mouse splenocytes were added to each well and stimulated with the recombinant protein purified in Method 2.4 at a concentration of 100 μg/ml. ConA was used as a positive control stimulant, and PBS was used as a negative control. Spot-forming cells (SFCs) were counted with an ELISPOT reader (AID ELISpot Reader iSpot, AID GmbH, Germany).

### 2.10 Flow cytometric analysis

Adaptive immunity primarily consists of humoral immunity and cellular immunity. Within cellular immunity, CD4^+^ and CD8^+^ T cells act in concert to mediate anti-infective immune responses. On 42 days, splenic lymphocytes from each group were divided into two aliquots and seeded into 12-well plates. One aliquot was stimulated with the corresponding protein, while the other remained unstimulated as a control. After 24 h, cells were harvested and stained in the dark with 1 μl of the following antibodies: FITC-conjugated anti-mouse CD4 antibody (BioLegend, USA) and PE-conjugated anti-mouse CD8 antibody (BioLegend, USA). Negative control tubes and single-color compensation tubes were included in parallel. The stained cells were incubated in the dark for 60 min, washed three times with PBS, and resuspended in 500 μl of PBS, blocking Fc receptors with Anti Mouse CD16/32 Antibody (Elabscience, CN). Finally, the cell suspensions were filtered through a mesh and analyzed by flow cytometry to determine the fluorescence intensity in each group.

### 2.11 Quantitative real-time PCR analysis of immune-related gene mRNA expression

The mRNA expression levels of interleukin-4 (IL-4), interleukin-6 (IL-6), tumor necrosis factor-alpha (TNF-α), and interleukin-10 (IL-10) in the spleen tissue of mice at 28 days and 42 days post-immunization were detected by means of quantitative real-time PCR (qRT-PCR). The primers for qRT-PCR were derived from previous studies (16). The amplification protocol was as follows: an initial 95 °C incubation for 30 s, succeeded by 40 cycles of 95 °C for 5 s and 60 °C for 30 s. The relative mRNA expression levels of the immune-related genes were computed using the 2^−ΔΔCt^ method and normalized to the expression of the housekeeping gene GAPDH (17).

### 2.12 Statistical analysis

Statistical analysis was performed using GraphPad Prism 9.0 to compare differences. All experiments were repeated three times. If the data followed a normal distribution, the homogeneity of variance was assessed, and one-way analysis of variance (ANOVA) was applied for comparisons among multiple groups. *Post hoc* pairwise comparisons were conducted using Dunnett's multiple comparison test for groups contrasted with a single control. If the homogeneity of variance was not achieved, Dunnett's T3 test (a modified version for unequal variances) was utilized for pairwise comparisons instead. Corresponding markers in the figures are denoted by asterisks: ^*^P < 0.05; ^**^P < 0.01; ^***^P < 0.001; ^****^P < 0.0001; ns: not significant. Statistical significance was set at *P* < 0.05.

## 3 Results

### 3.1 Evaluation and prediction of protein

**Figure 1:**
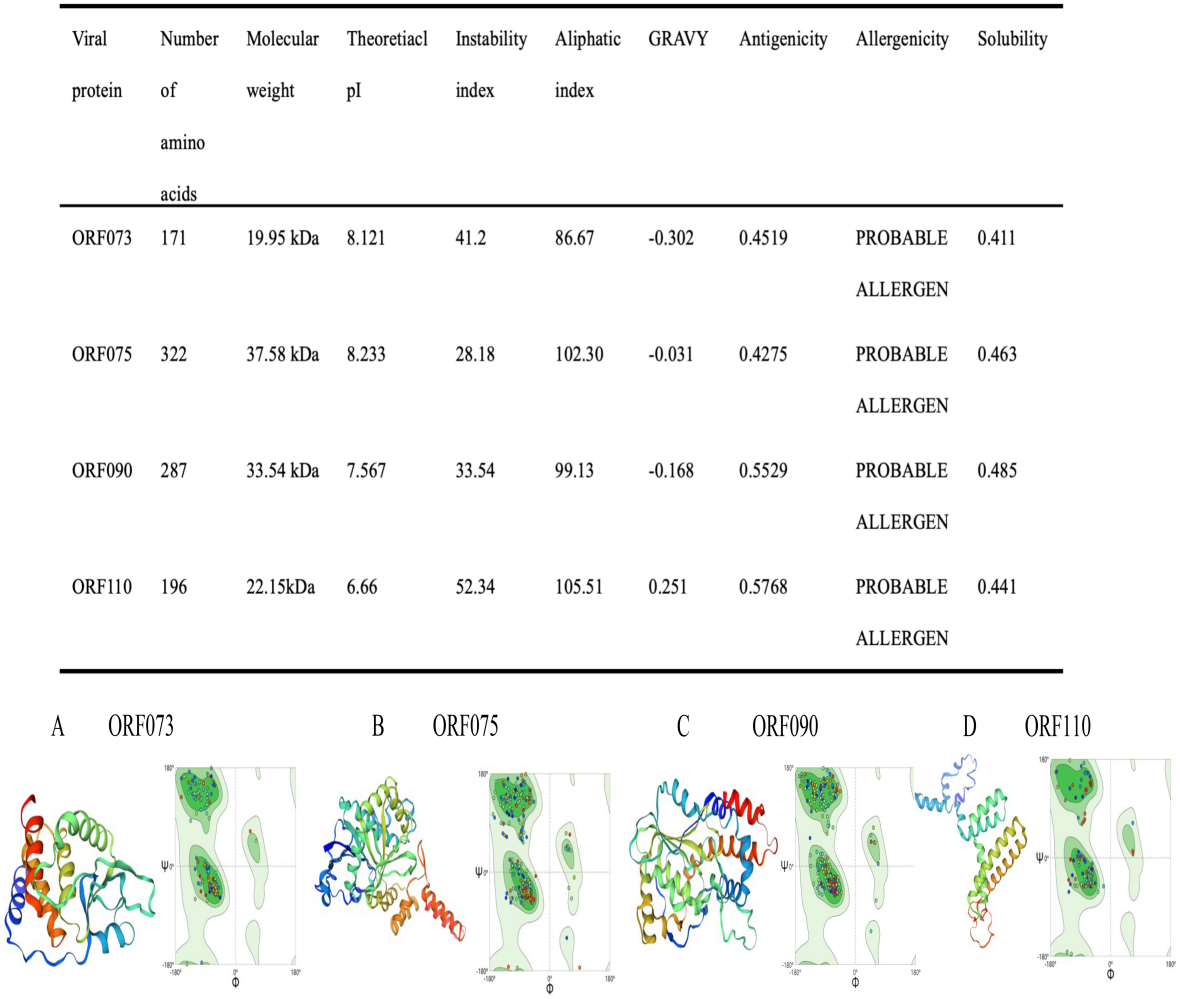
Evaluation and prediction results. (Table) Antigenic, allergenic and physiochemical assessments of the primary sequence of vaccine protein. **(A–D)** The three-level structure and Ramachandran plot of the vaccine predicted by the server.

Expasy Protparam server results suggest that the molecular weight of the four vaccine is approximately 19.95 kDa (ORF073), 37.58 kDa (ORF075), 33.54 kDa (ORF090), 22.15 kDa (ORF110) and its theoretical PI is 8.121 (ORF073), 8.233 (ORF075), 7.567 (ORF090), 6.66 (ORF110). The instability index is 41.2 (ORF073), 28.18 (ORF075), 33.54 (ORF090), 52.34 (ORF110). The grand average of hydropathicity (GRAVY) of the vaccine is −0.302 (ORF073), −0.031 (ORF075), −0.168 (ORF090), −0.251 (ORF110), demonstrating that it has a good hydrophilicity. The solubility value of the four vaccine is 0.411 (ORF073), 0.463 (ORF075), 0.485 (ORF090), 0.441 (ORF110; >0.4) according to the results of Protein-Sol server, which indicates a good solubility. The antigenicity value of the four vaccine is 0.4519 (ORF073), 0.4275 (ORF075), 0.5529 (ORF090), 0.5768 (ORF110), indicating that the four vaccine has an appropriate level of antigenicity, enabling it to induce a strong immune response. Furthermore, the four vaccine is non-allergenic and non-toxic based on results from AllerScreener and ToxinPred server (Figure 1).

### 3.2 Purification and expression of recombinant proteins

**Figure 2:**
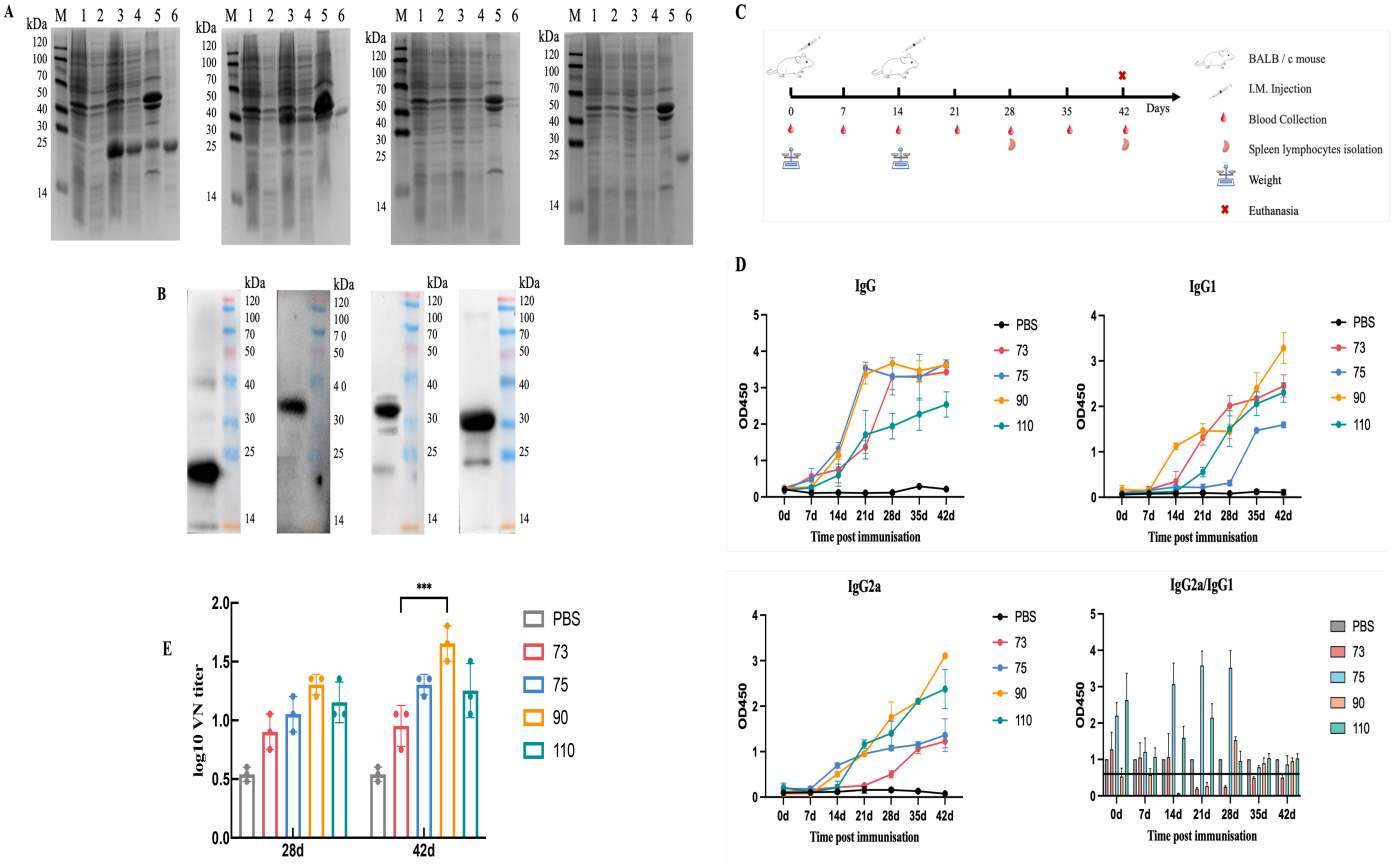
Protein expression, purification, and assessment of the immunization regimen in mice. **(A)** SDS-PAGE analysis of ORF073, ORF075, ORF090, ORF110: Lane M, protein labeling; Lane 1, uninduced pCold II BL21; Lane 2, uninduced; Lane 3, induced; Lane 4, supernatant of cells induced by ultrasound treatment; Lane 5, inducing cell precipitation after ultrasound treatment; Lane 6, purified protein. **(B)** Western blot analysis. **(C)** Timeline for immunization, blood, and tissue sampling schedules. **(D)** Changes of serum IgG1 and IgG2a antibody levels in mice. **(E)** Neutralizing antibody levels. Data were analyzed using two-way ANOVA to evaluate significant differences. ***P < 0.001; ns, no significant difference.

In the IPTG-induced cells (Figure 2A, lane 6), a single band with molecular weights of approximately 19.95 kDa (ORF073), 37.58 kDa (ORF075), 33.54 kDa (ORF090), and 22.15 kDa (ORF110) was observed for each protein, respectively. These values were consistent with the predicted molecular weights of the proteins. Additionally, the recombinant proteins were mainly present in the precipitate (containing inclusion bodies or cell debris; Figure 2A, lane 5). Subsequently, ORF073, ORF075, ORF090, and ORF110 were correctly expressed and exhibited strong reactivity in the prototype (Figure 2B) and utilized as subunit vaccines for the immunization experiments.

### 3.3 Expression levels of specific antibodies IgG, IgG1, and IgG2a in mouse serum

The immune program is shown in Figure 2C. After the first immunization, antibody levels in all groups remained low and showed no significant difference compared to the negative control group (*P* > 0.05). Following the second immunization, antibody levels in the immunized groups significantly increased compared to those observed after the first immunization (*P* < 0.01), indicating a continuous rise in antibody levels over time (Figure 2D).

An IgG2a/IgG1 ratio greater than 1 indicates a Th1-biased immune response, while a ratio less than 1 suggests a Th2-biased immune response (18). The IgG1/IgG2a ratios of mice immunized with ORF073 and ORF090 were less than 1 after the second immunization and subsequently approached 1. Meanwhile, the ratios for ORF090 and ORF110 were greater than 1 in the early stage and approached 1 after 28 days post-immunization, indicating a relatively balanced Th1/Th2 immune response.

### 3.4 Neutralizing antibody titer

In terms of the neutralizing antibody levels on 28 days and 42 days, we found that the ORF90 group exhibited the highest neutralizing antibody titer at both time points. Differences of varying degrees were observed among the groups (Figure 2E).

### 3.5 ELISA spot for measuring IFN-γ in mouse lymphocyte from spleen

**Figure 3:**
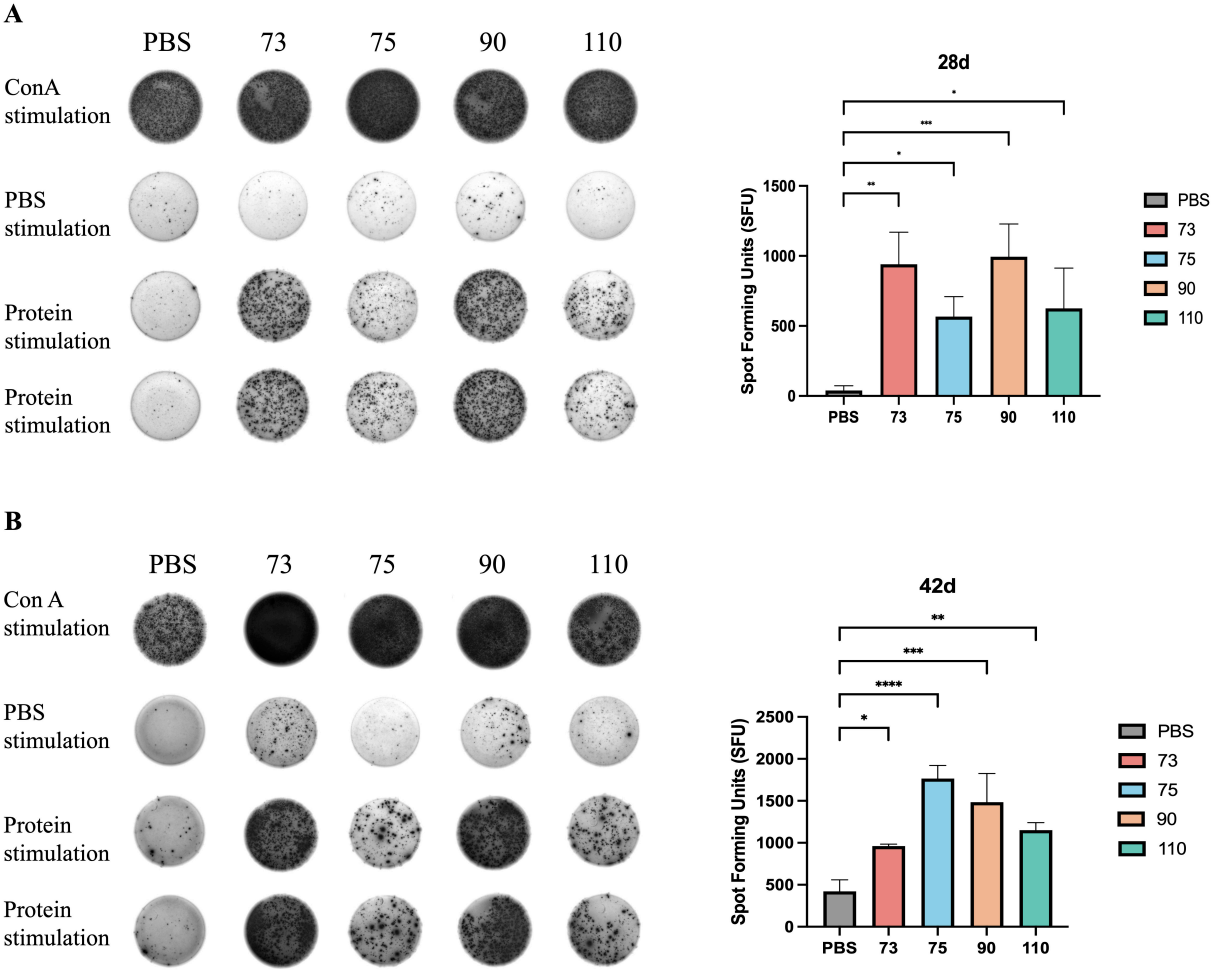
IFN-γ Secretion Levels in Mouse Splenocytes by ELISPOT detection. **(A)** Speck pattern of IFN-γ secretion in each group at 28 days of immunization. **(B)** Speck pattern of IFN-γ secretion in each group at 42 days of immunization. Data were analyzed using two-way ANOVA to evaluate significant differences. *P < 0.05; **P < 0.01; ***P < 0.001; ****P < 0.0001; ns, no significant difference.

Splenocytes were isolated and then stimulated with a mixture of recombinant proteins to evaluate the cellular immune response. As shown in Figures 3A, B, at 28 dpi, the frequency of IFN-γ-secreting splenocytes in the ORF75 and ORF90 groups was significantly higher than that in the PBS group (*P* < 0.001). When it came to 42 dpi, the secretion of IFN-γ by splenic lymphocytes in all immunized groups exhibited an increasing trend (Figure 3). These results indicate that the ORF75 and ORF90 groups can enhance T-cell-mediated cellular immune responses in mice.

### 3.6 Flow cytometry analysis of mouse splenic lymphocytes

**Figure 4:**
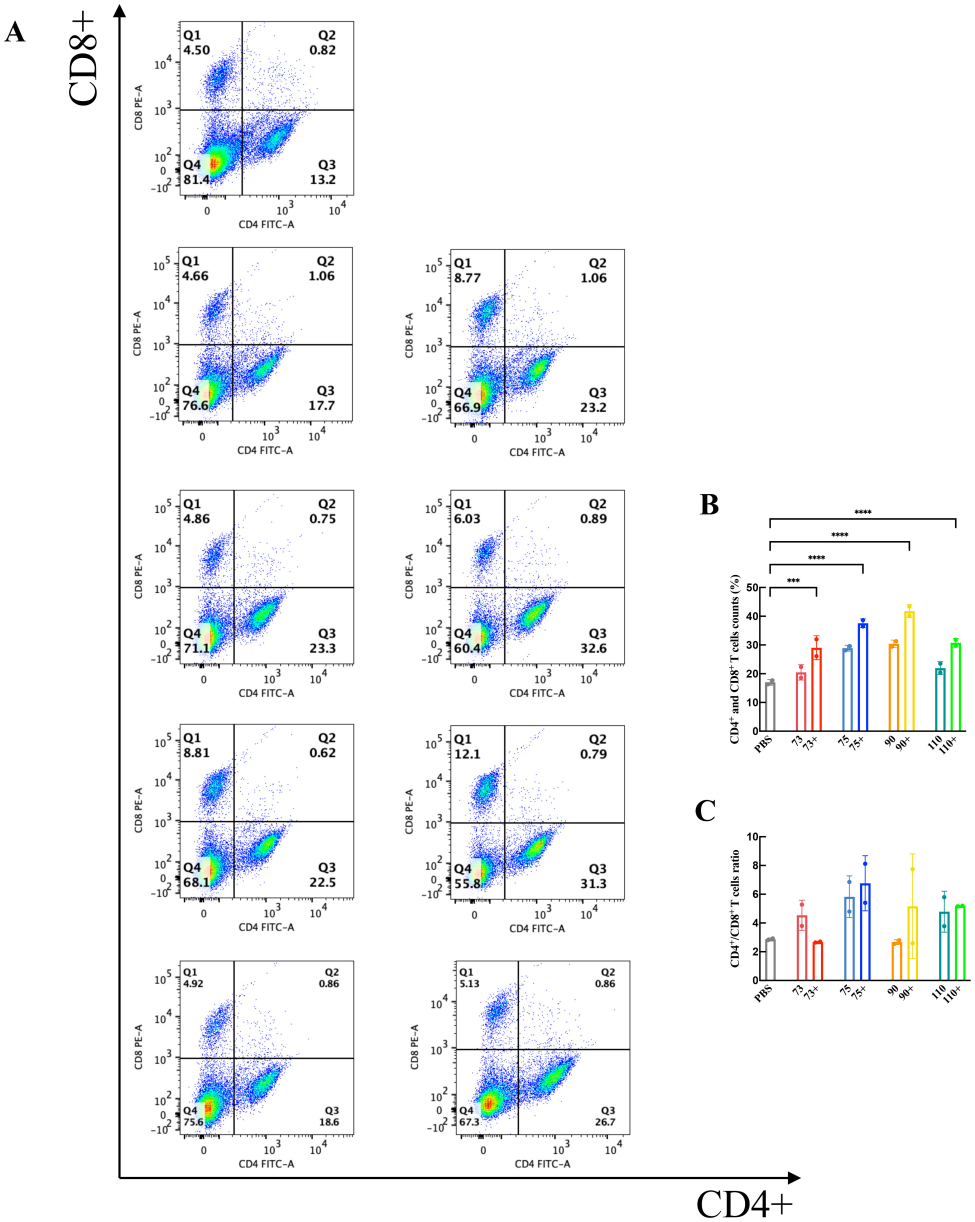
Flow cytometry analysis of splenic T-cell subsets. **(A)** Percentages of CD4^+^ and CD8^+^ T cells in immunized mice. **(B)** Proportions of CD4^+^ and CD8^+^ T cells within total splenic lymphocytes. **(C)** CD4^+^ and CD8^+^ ratio. Data were analyzed using two-way ANOVA to evaluate significant differences. ***P < 0.001; ****P < 0.0001; ns, no significant difference.

At 42 days post-immunization, splenic lymphocytes were isolated and subjected to flow cytometric analysis to evaluate the proliferation of CD4^+^ and CD8^+^ T cells (Figure 4). Compared with the PBS control group, the immunized groups displayed significantly higher counts of activated CD4^+^ and CD8^+^ T cells following antigen restimulation (*P* < 0.05). Furthermore, the percentage increase in CD4^+^ T cells was greater than that of CD8^+^ T cells, suggesting a vaccine-induced immune bias toward helper T cell (Th)-mediated responses. In summary, subunit immunization significantly enhanced the proportions of both CD4^+^ and CD8^+^ T cells.

### 3.7 mRNA expression levels of immune-related genes

**Figure 5:**
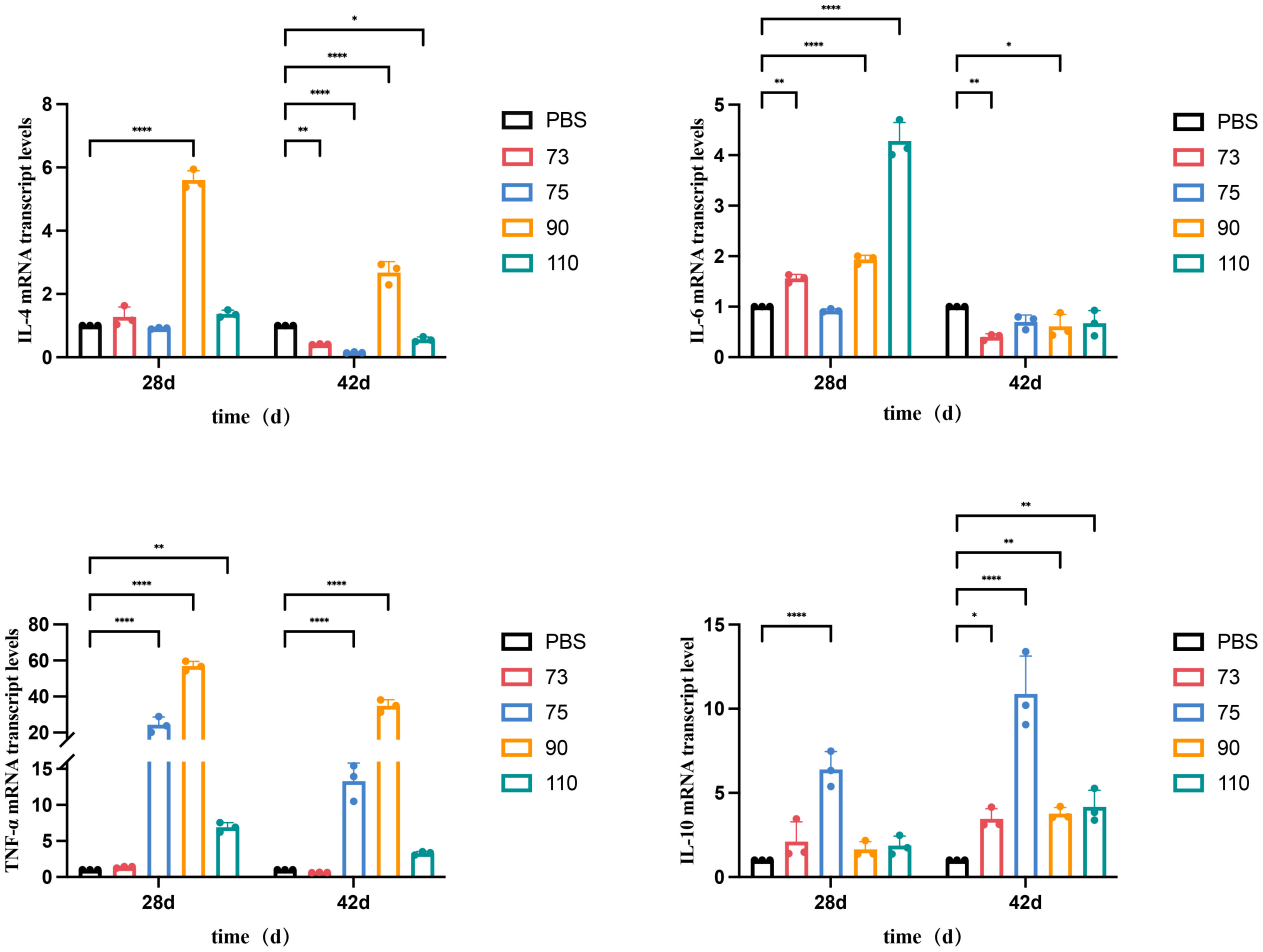
Detected the mRNA expression levels of immune related genes IL-4, IL-6, TNF-α, and IL-10 in mouse spleen lymphocytes using RT-PCR method. Data were analyzed using two-way ANOVA to evaluate significant differences. *P < 0.05; **P < 0.01; ****P < 0.0001; ns, no significant difference.

The mRNA expression levels of immune-related genes, in splenic lymphocytes were detected by qPCR. The mRNA expression level of IL-6 in the ORF110 group was significantly higher than that in the control group at 28 dpi (*P* < 0.01; Figure 5). The expression levels of TNF-α in the ORF75 and ORF90 groups were approximately 20-fold and 50-fold higher, respectively, compared to the control group (*P* < 0.01). Overall, protein immunization initially significantly upregulated the expression of pro-inflammatory cytokines like IL-6 and TNF-α, while the expression level of the anti-inflammatory cytokine IL-10 showed an upregulation trend over time.

## 4 Discussion

LSD represents a substantial threat to the livestock industry. It frequently manifests in epidemic patterns, giving rise to acute or subacute conditions in cattle and buffalo (19, 20). Cattle of all ages and breeds are susceptible, with young calves during the lactation period being particularly vulnerable (20). LSD is endemic across most African countries. Over the past decade, the disease has spread rapidly to numerous regions that were previously regarded as disease-free (14). In 2019, LSD made its first incursion into Asia (21). Recently, the World Organization for Animal Health (WOAH) has reported that lumpy skin disease (LSD), a notifiable disease to WOAH, broke out nearly simultaneously in France and Italy in June 2025 (https://www.woah.org/). At present, there is still uncertainty regarding which strain or variant is most appropriate for vaccine production (22). Regrettably, most inactivated Capripox vaccines have proven ineffective, offering merely transient protection (23). Internationally, relevant research data have demonstrated that certain inactivated vaccines can confer effective protection to cattle populations (20). Live Attenuated Vaccines provided cattle with substantial protection but also induced non-negligible side effects (20). A field study demonstrated that 22.9 and 2.31% of vaccinated cattle contracted the illness and succumbed to LSD, respectively. These findings underscore the importance of examining vaccination failures, including issues related to vaccine matching and the exploration of alternative vaccines (24). Another study in Ethiopia reported high morbidity and mortality rates among animals vaccinated annually, signifying the failure of LSD vaccines (25). The development of efficacious vaccines necessitates the screening of antigenic proteins encoded by the viral genome, which are capable of eliciting immune responses in the host organism (26). Thus, the development of effective vaccines is an urgent imperative.

Persistent efforts have been made to develop safer and more effective vaccine alternatives for the preventive immunization of animals against LSD. The current study evaluated the immunogenicity of recombinant ORF073, ORF075, ORF090, and ORF110 for the development of an LSD subunit vaccine. These proteins are homologous to poxvirus proteins involved in virion morphogenesis and assembly. Among them, transmembrane proteins, play a crucial role in the pathogenesis of LSDV by regulating viral structure and assembly, modulating immune system responses, and participating in nucleotide biosynthesis.

The development of subunit vaccines faces challenges including the selection of expression systems (among which the *Escherichia coli* system is widely used for pharmaceutical recombinant protein production), antigen production yield, and the choice of adjuvants in vaccine formulations. In this study, ORF073, ORF075, ORF090, and ORF110 were formulated with Freund's Adjuvant and used for immunization. As reported by Tursunov et al. (5), Freund's Adjuvant has been applied in LSDV subunit vaccine formulations, and their study demonstrated the compatibility and safety of this adjuvant when used with LSDV preparations.

Subsequently, we explored the immunogenic potential of the protein subunits using a mouse model. Our results demonstrated that high-titer antibodies could be induced with just two injections. Subsequently, to assess the Th1/Th2 bias in the immune response elicited by the recombinant proteins, an antibody isotyping assay was employed to measure the epitope-specific IgG1 and IgG2a antibody titers (27). The IgG2a/IgG1 ratio in ORF075-immunized mice was greater than 1 during the middle stage of the immunization process, indicating a Th1-biased immune response. In contrast, the IgG1/IgG2a ratio in mice immunized with other recombinant proteins approached 1, suggesting a relatively balanced Th1/Th2 immune response.

Cytokines, as soluble mediators of the immune system, play a critical role in antiviral defense. In capripoxvirus infections, virus-infected macrophages release pro-inflammatory chemokines and cytokines, which activate infected yet quiescent lymphocytes and macrophages (28).

The regulation of immune cell subsets is an important aspect of the immune response. The type of cell subsets is primarily determined by cytokines secreted by migrating dendritic cells and other innate immune cells in the microenvironment of draining lymph nodes. In this study, the results of cytokine secretion by stimulated splenocytes were analyzed. Under the same antigen dose and adjuvant, the levels of IL-4, IL-6, and TNF-α in immunized animals were significantly elevated, which was consistent with previous studies (29). Subsequently, these cytokine levels decreased and stabilized. Another study reported an increase in TNF-α levels in buffalo infected with LSDV, 21 days after the appearance of skin nodules (30). The elevated levels of Th1 cytokines IFN-γ and TNF-α during LSD infection suggest that increased IFN-γ expression in subclinical cattle may inhibit viral proliferation until the viral load is too low to cause disease.

Cellular immunity plays a pivotal role in vaccination by generating memory and effector T cells, which are essential for long-term immune protection. Upon viral infection, the host mounts a coordinated defense involving both innate and adaptive immune responses (31). Among these, T cells serve as key mediators of antiviral immunity. CD4^+^ T cells orchestrate immune responses by facilitating B-cell antibody production and regulating other immune cell functions. These cells not only initiate primary immune responses against pathogens but also differentiate into Th1 and Th2 subsets, thereby shaping adaptive immunity. In contrast, CD8^+^ T cells directly eliminate infected cells through cytotoxic activity. Notably, the cytotoxic capacity of T lymphocytes can be enhanced by upregulated CD8 expression (32). In addition, flow cytometry and ELISpot results indicate that Subunit vaccine effectively induces strong LSDV specific cellular immune responses.

In summary, we successfully constructed four subunit vaccines, ORF073, ORF075, ORF090, and ORF110, by utilizing specific proteins derived from LSDV. These vaccines stimulated robust cellular and humoral immune responses in mice, resulting in a significant increase in high levels of IgG titers. Additionally, the ORF75 and ORF90 vaccines elicited a more pronounced increase in T-cell-mediated cellular immune responses, as evidenced by elevated IFN-γ levels. These findings significantly contribute to the selection of antigens and the optimization of vaccination strategies to enhance the efficacy of LSD vaccines. However, this study has some limitations. Specifically, only the immune response induced by the vaccine in mice was evaluated. Therefore, it is necessary to evaluate the protective efficacy of the vaccine prototype in bovines in future experiments, this is our next research work.

## Data Availability

The original contributions presented in the study are included in the article/supplementary material, further inquiries can be directed to the corresponding authors.
